# Effect of Quercetin on Haematobiochemical and Histological Changes in the Liver of Polychlorined Biphenyls-Induced Adult Male Wistar Rats

**DOI:** 10.1155/2013/960125

**Published:** 2012-10-01

**Authors:** Kandaswamy Selvakumar, Senthamilselvan Bavithra, Sekaran Suganya, Firdous Ahmad Bhat, Gunasekaran Krishnamoorthy, Jagadeesan Arunakaran

**Affiliations:** ^1^Department of Endocrinology, Dr. ALM Post Graduate Institute of Basic Medical Sciences, University of Madras, Chennai 600113, India; ^2^Department of Biochemistry, Asan Memorial Dental College & Hospital, Asan Nagar, Chengalpattu 603105, India

## Abstract

Polychlorinated biphenyls exposure damages the rat liver cells. Hematological parameters such as hemoglobin, packed cell volume, red-blood cells, white-blood cells, neutrophils, platelet counts, and RBC indices were significantly decreased. Polymorphs, eosinophil counts, and erythrocyte sedimentation rate were significantly increased. Serum liver enzymes such as aspartate transaminase, alanine transaminase, alkaline phosphatase, and gamma-glutamyl transferase were increased by PCBs treatment. Serum lipid profiles such as cholesterol, triglycerides, low-density lipoproteins and very-low-density lipoproteins were increased in PCBs-treated rats. High-density lipoprotein, total protein, albumin, globulin levels, and albumin/globulin ratio were also decreased after PCB exposure. Then levels of sodium, potassium, chloride, and bicarbonate were also altered. Serum glucose levels were increased along with total bilirubin after PCBs exposure. Simultaneous quercetin supplementation significantly protected the PCBs-induced changes of hematobiochemical parameters. Thus, quercetin shows a protective role against PCBs-induced alterations in the hematological and biochemical parameters.

## 1. Introduction

Polychlorinated biphenyls (PCBs) are industrial chemicals used in plasticizers, surface coatings, inks, adhesives, flame retardants, pesticide extenders, paints, and microencapsulation of dyes for carbonless duplicating paper. Because PCBs resist both acids and alkalis and are relatively heat-stable, they have been used in dielectric fluids in transformers and capacitors. Further environmental contamination may occur from the disposal of old electrical equipment containing PCBs.Their characteristic low solubility, contribute to its ability to bioconcentrate which leads to bioaccumulation. PCBs have been regulated as food contaminants and in foodstuffs. They are generally higher chlorinated with high resistance to metabolic breakdown [1]. The production of PCBs peaked in the 1970s and has steadily declined thereafter as many countries throughout the world have banned their use or limited their production. Nevertheless these compounds remain in use today in our environment and represent a potential human health hazard [2]. PCBs are environmental toxicants associated with numerous adverse health effects, through widespread bioaccumulation in the biosphere and bioconcentration in the food chain [3]. PCB-induced toxic manifestations are associated with the production of free radicals [4]. Oxidative impairment occurs when generation of ROS overrides the ability of the antioxidant system to neutralize ROS subsequently leading to both an increase in oxidative processes and a decrease in antioxidant defenses.

The flavonoids are a large group of naturally occurring compounds that are found in plants and are frequently consumed as part of the human diet. Flavonoids are receiving much attention now a days for their potential pharmacological properties. It has also been reported that quercetin a polyphenolic flavonoid possesses antitumoral [5], anti-allergic [6], vasorelaxative [7], antiischemic [8] and anti-inflammatory activity [9]. The mechanisms underlying these effects were thought to come from their antioxidant property. The antioxidant activity of flavonoids has been demonstrated by their ability to inhibit enzymes such as lipoxygenase, cyclooxygenase [10], along with chelating metal ions [11], and scavenging free radicals [12]. Inspite of its role as free radical scavenger, there was no clear report in the antioxidant effect of quercetin on the PCB-induced rats.

Volatile compounds like halothane and enflurane may increase the serum activities of aspartate aminotransferase (AST), alanine aminotransferase (ALT), and alkaline phosphatase (ALP), and affect biochemical and hematological parameters in laboratory animals [13–15]. Accumulation of volatile metabolites and other free radicals in hepatic cellular components may increase cellular degeneration of the liver. High levels of these enzymes are indicators of hepatic damage both human and experimental animals [16]. Several supplements have been used to protect the liver from damage including administration of antioxidants such as *β*-carotene [16], vitamin C [17], vitamin E, and so forth. Previous studies in our laboratory demonstrated the protective role of quercetin on PCB-induced oxidative stress and apoptosis in hippocampus [18], cerebellar [19] and cerebral cortical dopaminergic receptor expression [20]. The role of quercetin in protecting the hematological parameters has not yet been studied.

Hence, the present study was designed to assess the impact of quercetin on hematological and biochemical parameters in the blood of PCBs-exposed rats including liver histology. The parameters are on blood and serum enzyme activities, protein, lipid, electrolytes, and nonnitrogenous compounds.

## 2. Materials and Methods

### 2.1. Chemicals

Aroclor 1254 (PCBs) was purchased from M/s. Chem Services, West Chester, PA (USA). Quercetin and all other molecular grade chemicals were purchased from M/s. Sigma-Aldrich Pvt. Limited (USA).

### 2.2. Experimental Design

 Healthy adult male albino rats of Wistar strain, *Rattus norvegicus,* weighing about 180–200 g (90 days) were used in the present study. The study protocol was reviewed and approved by the institutional ethical committee (Ref no. IAEC no: 01/01/11). The animals were housed in clean polypropylene cages, maintained in air-conditioned animal house with constant photoperiod of 12 h light/dark cycle for 30 days. They were fed with pellet diet (Gold Mohur Ltd., Mumbai, India) and drinking water *ad libitum.* The animals were divided into four groups, 6 in each. They were treated by interaperitoneal (i.p.) for group I (control) corn oil as vehicle; by gavage for group II quercetin 50 mg/kg of body mass dissolved in 0.9% physiological saline [18]; by interaperitoneal (i.p.) for group III Polychlorinated biphenyl (Aroclor 1254 dissolved in corn oil) 2.0 mg/kg of body mass [21]; group IV—PCB i.p. and simultaneous supplementation of quercetin by gavage treatment [18].

On the 30th day, the animals were sacrificed. Blood samples were collected for biochemical investigations and for analyzing hematogical parameters, they were collected in tubes containing EDTA. For biochemical investigations, the blood samples were centrifuged at 5000 rpm for 20 min at 4°C; the serum obtained was stored at −20°C until analysis. Liver tissues were also collected for histological analysis. Whole blood samples were collected separately for the determination of complete haemogram using Sysmex KX-21, TRANSASIA. Biochemical parameters were determined using Konelab20—fully automated biochemical analyzer, Thermo scientific, Finland and the electrolytes (Sodium, Potassium, Chloride, and Bicarbonate) were analyzed using Blood gas analyzer, Siemens Diagnostics 348. 

### 2.3. Evaluation of Complete Blood Count

Complete blood count includes hemoglobin (Hb), packed cell volume (PCV), total red blood corpuscles (TCRBC), total count of white blood cells (TCWBC), differential count (DC), platelets count, RBC indices such as mean corpuscular volume (MCV), mean corpuscular haemoglobin (MCH), and mean corpuscular haemoglobin concentration (MCHC) were analyzed by Sysmex KX-21, TRANSASIA, a fully automated 3-part differential hematology analyzer. Erythrocyte sedimentation rate (ESR-(1/2) Hr and 1 Hr) was estimated by Westergren method  (1926).

### 2.4. Peripheral Blood Smear

#### 2.4.1. Preparation of a Thin Blood Flim

A thin blood film was made by spreading a drop of blood evenly across a clean grease-free slide, using a smooth-edged spreader. Leishman's stain (Powdered Leishman's Stain 0.15 gm, Acetone-free methyl alcohol 133 mL) was used for the staining of dried blood smear. Few drops of Leishman's stain were added on the slide and kept for 2 mins. Then twice the amount of water was added and left undisturbed for 7–10 mins. The excessive stain was flushed off with distilled water, and the slide was completely air dried for 2-3 mins.

### 2.5. Determination of Biochemical Parameters

In the sera of control, and experimental grouped rats, the following parameters were determined: Glucose, BUN, Urea, Creatinine, Uric acid, Enzymes (AST, ALT, ALP, GGT, Amylase, Lipase, and CPK), Protein Metabolism (T. Protein, Albumin, Globulin, and A/G ratio), and Lipid Profile (T. Cholesterol, Triglycerides, HDL Cholesterol, LDL Cholesterol, and VLDL Cholesterol) (Table 1). Konelab20—fully automated biochemical analyzer (Thermo scientific, Finland) was used to determine the concentrations of all these biochemical parameters in rats' sera and electrolytes (Sodium, Potassium, Chloride, and Bicarbonate) were estimated by ion selective electrode (ISE) by using blood gas analyzer Siemens Diagnostics 348. 

### 2.6. Histological Studies

 The liver tissues taken for histological analysis were separated, cleaned and diced into 0.5 cubic cm in volume. Then they were fixed in buffered formaldehyde (4% final concentration) prepared in phosphate buffer saline (PBS) (pH 7.4) by completely immersing them and left overnight at room temperature (8–12 hrs). The buffered formaldehyde was drained and 70% ethanol was added and kept at 4°C until paraffin blocks were made. Later, the tissue sections were processed for paraffin sectioning, and tissue blocks were made. The blocks were cut into 5 *μ*m thickness using rotary microtome and stained with haematoxylin and eosin [22]. The liver cellular morphology was analyzed using Nikon Advanced Research Microscope ECLIPSE 80i.

### 2.7. Statistical Procedure

 Values are given as mean ± standard error mean (SEM). The statistical analysis of biochemical parameters were conducted using the SPSS 17 software. GraphPad Prism 5 was used for statistical analyses and graphics (GraphPad Software, Inc., La Jolla, CA, USA). The variance homogeneity was analyzed using the Students Newman's Kuel's (SNK). In order to compare mean values, the data were subjected to one-way ANOVA analysis, followed by Dunn's post hoc test. The critical significance level was set as *P* < 0.05.

## 3. Results

### 3.1. Body Weight of PCBs-Exposed Adult Rats

**Figure 1:**
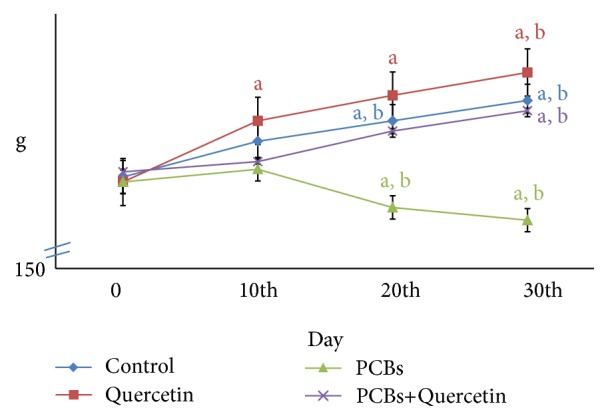
Effect of quercetin on body weight of PCBs-exposed adult rats. Each value represents mean ± SEM of 6 animals. Statistical significance at *P* < 0.05. a: Control versus others; b: Quercetin versus PCB, PCB+Quer.

The effect of PCBs on body weight of rats was shown in Figure 1. No mortality was observed in any of the experimental groups. Maximum weight gain was observed in the quercetin-administered rats when compared to all other groups. There was a gradual and significant (*P* < 0.05) decrease in body weight of PCBs treated rats when compared to control and quercetin-treated rats.

### 3.2. Effect of Quercetin on Hematological Parameters of PCBs-Exposed Adult Rats

Hematological parameters and their comparisons among all groups are presented in Table 2. Compared to that of the control, the values of Hb, PCV, RBC, WBC, neutorphils, and platelet count were significantly decreased by PCB treatment. Polymorphs, eosinophils counts, and erythrocyte sedimentation rate were significantly increased by PCB treatment, but the values of Hb, PCV, RBC, WBC, neutorphils, and platelet rates were significantly increased with simultaneous administration of quercetin along with PCB. However, the MCV value was significantly decreased in PCB-treated rats, however MCH, MCHC values did not alter in PCB- or quercetin-treated rats.

### 3.3. Effect of Quercetin on Peripheral Blood Smear of PCBs-Exposed Adult Rats (100x)

**Figure 2:**
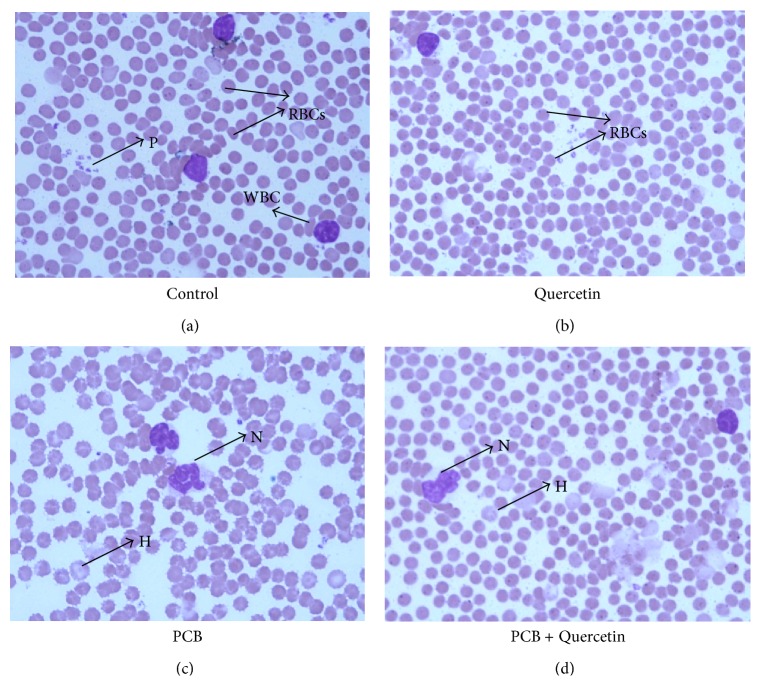
Effect of quercetin on Peripheral blood smear of PCBs-exposed adult rats (100X). Peripheral smear study of PCBs-exposed and other group (Leishman's Stain, Nikon Eclipse 80i, 100x oil immersion). Microphotograph of the blood smear of control (a), quercetin (b), PCB (c) and PCB+Quercetin (d) rats. (a), (b), and (d) show the Normochromo normocytic RBCs; leucocytes and platelets are adequate. PCB (c). Total number of RBS are less in number and it shows hypochromo microcytic RBCs, leucocytes are normal, platelets are less in numbers. P: platelets, H: hypochromic microcytic cells, N: neutrophils.

Figure 2 shows peripheral smear study of PCBs-exposed and other group (Leishman's Stain, Nikon Eclipse 80i, 100x oil immersion). Microphotograph of the blood smears of Control (a), Quercetin (b), PCB (c), and PCB+Quercetin (d) rats were taken. (a), (b), and (d) show a Normochromo normocytic RBCs whereas Leucocytes and Platelets were adequate, PCB (c). Total numbers of RBCs were less in number and it showed Hypochromo microcytic RBCs, leucocytes were normal, Platelets were less in number.

### 3.4. Effect of Quercetin on Serum Enzyme Activities (Liver, Digestive, and CPK) of PCBs-Exposed Adult Rats

**Figure 3:**
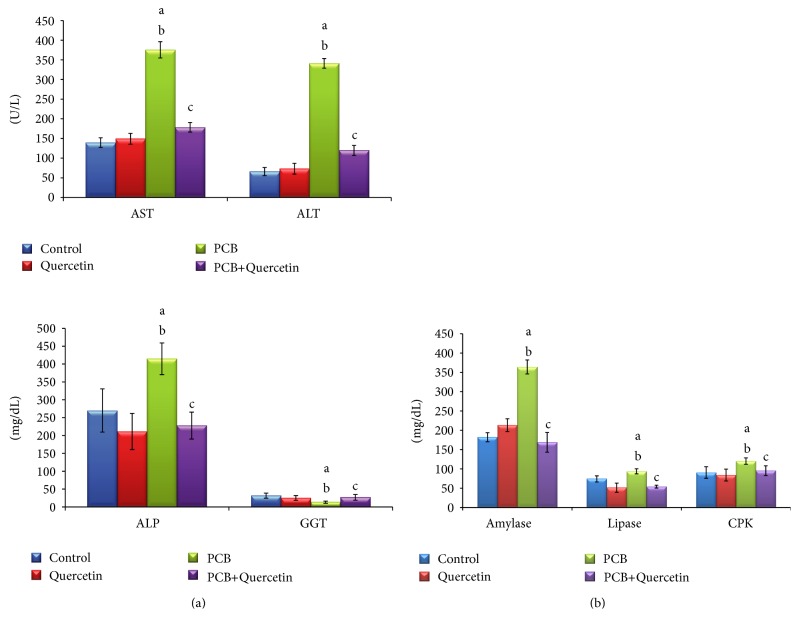
(a) Effect of quercetin on serum liver enzyme activities of PCBs-exposed adult rats. Each bar represents mean ± SEM of 6 animals. Statistical significance at *P* < 0.05. a: control versus others; b: Quercetin versus PCB, PCB+Quer; c: PCB versus PCB+Quer. AST: aspartate transaminase, ALT: alanine transaminase, ALP: alkaline phosphatase, GGT: gamma glutamyl transaminase. (b) Effect of quercetin on serum amylase, lipase, and CPK activities in PCBs-exposed adult rats. Each bar represents mean ± SEM of 6 animals. Statistical significance at *P* < 0.05. a: control versus others; b: quercetin versus PCB, PCB+Quer; c: PCB versus PCB+Quer CPK: creatinine phosphokinase.

The activities of liver enzymes such as AST, ALT, ALP, and GGT were significantly increased by PCB treatment, but decreased in the simultaneous quercetin-treated rats (Figure 3(a)). The activities of enzymes such as amylase, lipase and creatinine phosphokinase were represented in Figure 3(b). The activities of all the enzymes were increased in PCBs-treated whereas simultaneous quercetin treatment retrieved the enzyme activities as that of control.

### 3.5. Effect of Quercetin on Liver Histology in PCBs-Exposed Adult Male Rats (Haematoxylin and Eosin Staining, (a, b, c, d 40x) and (A, B, C, D 100x)

**Figure 4:**
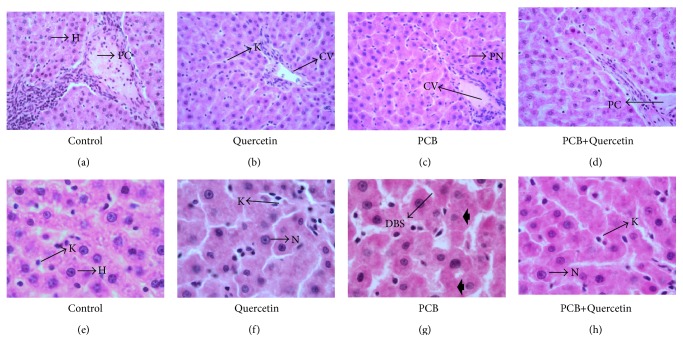
Liver histology in control, quercetin-, PCB, and PCB+Quercetin-treated adult male rats (Haematoxylin and Eosin staining, ((a), (b), (c), (d) 40x) and ((e), (f), (g), (h) 100x). Photomicrograph of the liver cells ((e)-(f)) Liver sections of control (e) and quer-treated (f) rats showing normal histological appearance of the liver, including portal canal (PC), hepatocytes (H), centrally located nuclei (N), and Kupffer cell (K). (g) Section of rat liver treated with PCB revealed considerable number of damaged hepatocytes (H) that have lost their characteristic appearance, with appearance of Pyknotic Nuclei (PN), apoptotic hepatocytes (bolded arrow) and dilation in blood sinusoids (DBS). (h) Liver section of the rat treated with PCB supplemented with quer demonstrated restoration of normal arrangement of hepatocytes, although few damaged hapatoctes were also observed. (Haemotoxylin and Eosin staining, 40x and 100x).

Figure 4 shows the histological appearance of liver in control, quercetin (drug control), PCB-administrated, and PCB along with simultaneous supplementation of quercetin in the liver of adult rats. *Control group:* the liver sections of the control group showed normal lobular architect with hepatocyte arranged in cords encircling the central canal, which also showed normal histological features such as, large polygonal cells with prominent round nuclei and few spaced hepatic sinusoids arranged inbetween the hepatic cords with fine arrangement of Kupffer cells around hepatic portal canal (Figure 4(a) 40x; Figure 4(e) 100x).

*Quercetin-treated group: *The liver sections of quercetin-treated group showed normal polygonal cells around the portal canal and sinusoid showed a layer of fenestrated endothelial cells. Kupffer cells showed a normal liver architecture with mild infiltration of lymphocytes and few macrophages in the periportal zones and no apoptosis was seen (Figure 4(b) 40x; Figure 4(f) 100x). *PCBs-treated group: *sections from the liver exposed to PCB showed moderate infiltration of chronic inflammatory cells mainly lymphocytes along with increased fibroblasts in the periportal and centrilobular zones. Few hepatocytes in these regions showed apoptotic changes such as nuclear shrinkage, pyknotic Nuclei (PN), Karyolysis, and Dilation in blood sinusoids (DBS). (Figure 4(c) 40x; Figure 4(g) 100x). *PCBs+Quercetin-treated group: *sections from the liver showing mild periportal and centrilobular chronic inflammatory infiltrate composed of lymphocytes and few macrophages and dilated central veins seen in few lobules, when compared to PCB-treated rats. At a higher magnification, restoration of normal hepatocytes with centrally located nuclei and a very few degenerated hepatocytes were seen (Figure 4(d) 40x; Figure 4(h) 100x).

### 3.6. Effect of Quercetin on Serum Lipid Profiles of PCBs-Exposed Adult Rats

**Figure 5:**
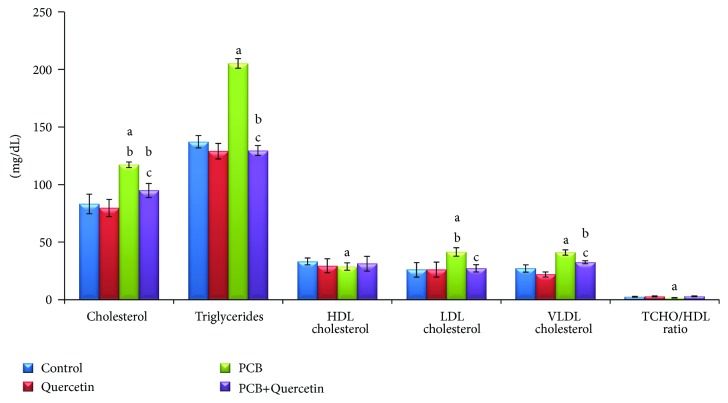
Effect of quercetin on serum lipid profiles of PCBs-exposed adult rats. Each bar represents mean ± SEM of 6 animals. Statistical significance at *P* < 0.05. a: control versus others; b: quercetin versus PCB, PCB+Quer; c: PCB versus PCB+Quer.

The lipid profiles parameters are depicted in Figure 5. The concentrations of CHOL,TGL, LDL and VLDL cholesterol in the PCB group were statistically increased in the PCBs- treated group whereas simultaneous quercetin administered group shows significant decrease in CHOL, TGL, LDL, and VLDL value was observed. HDL cholesterol value was slightly decreased in PCBs-treated than the control animals. There was a total decrease of CHOL/HDL ratio in the PCB-treated group and no significant change in quercetin alone treated group.

### 3.7. Effect of Quercetin on Serum Protein Metabolism of PCBs-Exposed Adult Rats

**Figure 6:**
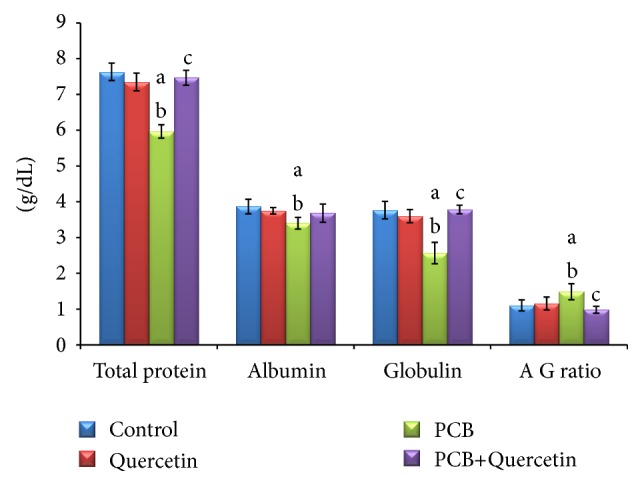
Effect of quercetin on serum protein metabolism of PCBs-exposed adult rats. Each bar represents mean ± SEM of 6 animals. Statistical significance at *P* < 0.05. a: control versus others; b: quercetin versus pCB, PCB+Quer; c: PCB versus PCB+Quer.

Total protein, albumin, globulin, and A/G ratio were assessed to diagnose protein metabolism. The changes in total and individual protein levels are presented in Figure 6. In PCB-treated rats, there was a significant decrease in total protein, albumin, globulin levels, and A/G ratio when compared with the control group. On simultaneous treatment of quercetin along with PCB the total protein, albumin, and globulin levels were brought back to normal.

### 3.8. Effect of Quercetin on Selected Serum Electrolytes of PCBs-Exposed Adult Rats

**Figure 7:**
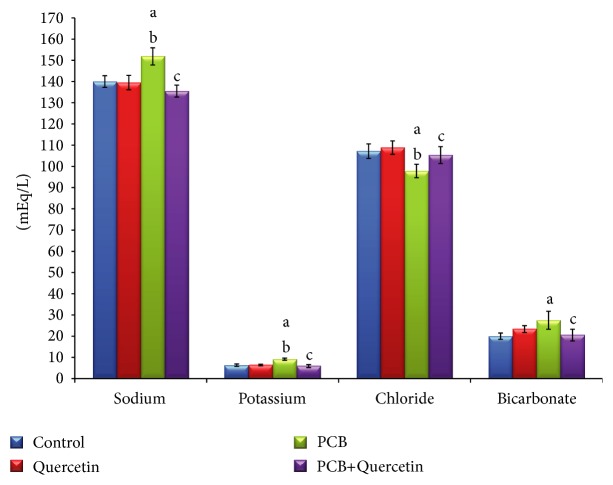
Effect of quercetin on selected serum electrolytes of PCBs-exposed adult rats. Each bar represents mean ± SEM of 6 animals. Statistical significance at *P* < 0.05. a: Control versus others; b: Quercetin versus PCB, PCB+Quer; c: PCB versus PCB+Quer.

The levels of sodium, potassium, chloride, and biocarbonate were assessed in treated and control rats to detect any imbalance in electrolytes which could have occurred due to PCB treatment is represented in Figure 7. There was a significant increase in serum sodium level and significant decrease in chloride level of PCB-treated rats whereas the potassium and bicarbonate levels did not alter. Simultaneous PCB+Quercetin administered rats showed significant decrease in sodium levels when compared with control and quercetin treated rats.

### 3.9. Effect of Quercetin on Serum Nonprotein Nitrogenous Compounds of PCBs-Exposed Adult Rats

**Figure 8:**
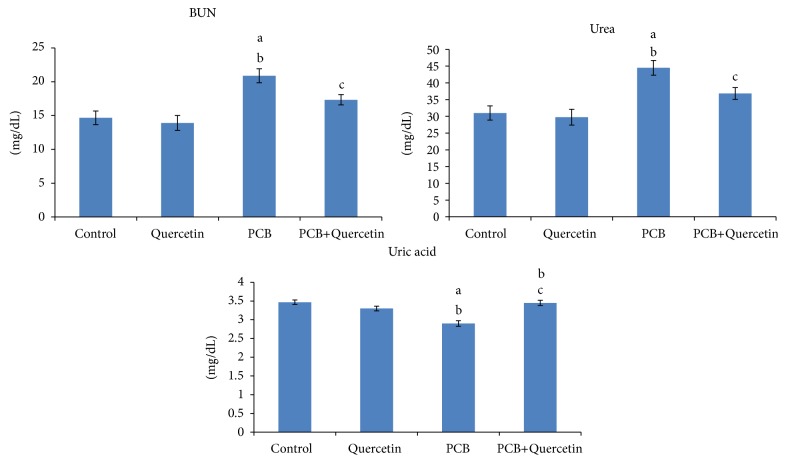
Effect of quercetin on serum non-protein nitrogenous of PCBs-exposed adult rats. Each bar represents mean ± SEM of 6 animals. Statistical significance at *P* < 0.05. a: Control versus others; b: Quercetin versus PCB, PCB+Quer; c: PCB versus PCB+Quer.

The values of serum BUN, urea, and uric acid are depicted in Figure 8. The levels of BUN and urea were significantly increased in PCBs-treated rats whereas the uric acid levels did not alter. Quercetin-alone-treated group did not show any significant alteration. 

### 3.10. Effect of Quercetin on Serum General Biochemical Parameters of PCBs-Exposed Adult Rats

The other general biochemical parameters like glucose, creatinine, and total bilirubin are depicted in Table 3. Significant increase in serum glucose, creatinine, and total bilirubin values were observed in PCB treated rats compared to control. Quercetin-alone-treated rats did not show any alteration in any of the above parameters.

## 4. Discussion

Polychlorinated biphenyls (PCBs) are considered as “persistent organic pollutants” that accumulate in individuals and magnify in the food chain. PCB bioaccumulation can lead to reduced infection lighting ability, increased rate of autoimmunity, cognitive problems, behavior-associated problems, and hypothyroidism [23]. PCBs can cause liver injury, especially after chronic exposures. PCBs are metabolized by the cytochrome P450 microsomal system in the liver. Liver plays a central role in xenobiotic metabolism. PCBs exposure leaves the liver prone to xenobiotic-induced injury. However, the mechanism that leads to PCBs hepatotoxicity is not very clear. It has been generally accepted that PCBs is transformed by either oxidative or reductive pathways, depending on tissue oxygen concentrations [21]. Once PCBs enter in the biological system it is transformed into primary and secondary metabolites. These intermediary metabolites, responsible for the hepatotoxic effect of PCBs, may bind to cellular macromolecules and react with free amino groups of proteins, hence the macromolecules may lose their physiological functions [24] or stimulate hepatocytes to produce more toxic metabolites [25]. The intermediary metabolites produced during biotransformation of volatile compounds are held responsible for hepatotoxicity and the increase of serum activities of liver enzymes. They may cause cellular damage by covalent binding to cellular components such as enzymes, nucleic acids, and proteins or by any another mechanisms. Damage of cellular components may play an important role in death of liver cells [13, 24]. Consequently, serum levels of AST, ALT, and ALP enzymes would increase due to its release. High serum levels of AST and ALT are usually indicative of liver damage in animals [25, 26] and humans [27, 28]. Liver-ALP is mobilized most rapidly into blood and its levels in serum may increase at early period of liver damage. High ALP serum level is usually indicative of cholestasis which may also result in a progressive liver disease—biliary cirrhosis. In the present study also increased serum levels of all parameters were observed, indicative of liver damage.

Amylase is an enzyme that helps to digest the glycogen and the starch. It is produced mainly by exocrine pancreas and salivary glands. This is determined necessarily in diagnosis and control of acute or chronic pancreatitis. It can also reflect biliary or gastrointestinal disease and other upheavals. Lipase [LPS] is a pancreatic enzyme necessary for the absorption and digestion of nutrients that catalyzes the hydrolysis of glycerol esters of fatty acids. Estimation of LPS is used for diagnosis of pancreatic diseases such as acute and chronic pancreatitis and obstruction of the pancreatic duct [29]. Increased level of amylase and lipase were observed in the PCBs treated animals. Creatine kinase is a cellular enzyme widely distributed in the tissues of the body. Its physiological role is associated with adenosine triphosphate (ATP) generation for contractile or transport systems. Elevated CK values are observed in PCB-treated group due to the increased lipid metabolism which forms the main cause for myocardial infarction.

The light microscopic investigations also showed many histological abnormalities in the liver of PCBs-exposed rats including disruption of hepatic cords and dilated blood sinusoids. Many hepatocytes showed apoptotic indicative characteristics like, karyomegaly and pyknotic nuclei. On the other hand, liver sections of the rat exposed to PCBs and simultaneously supplemented with quercetin demonstrated restoration of normal arrangement and reduced apoptosis of hepatocytes.

Quercetin as a bioflavonoid may play an important role in physiological reactions such as mixed oxidation function involving incorporation of oxygen into a biochemical substrate. In addition, quercetin is also considered as the most important antioxidant in extracellular fluids and its antioxidant function has been shown to efficiently scavenge superoxide, hydrogen peroxide, lipid peroxidation, and also protein carbonyl compound.

Reduction in Hb, PCV, RBC, TC, Platelets levels, and RBC indices such as MCV, MCH, and MCHC (Table 2) were observed following exposure of PCBs in rats of groups III and IV which revealed microcytic hypochromic anemia. This hematological alteration might be due to PCBs effect on activity of aminolevulinic acid dehydratase (ALAD), a key enzyme of heme synthesis. Balani et al. [30], proved that lead toxicity inhibits the conversion of coproporphyrinogen III to protoporphyrin IX leading to reduction in hemoglobin production and shortened life span of erythrocytes. Increase fragility and progressive destruction of RBCs due to binding of free radicals produced by PCBs could be another reason for decreased haematological values [30]. Similarly significant decrease in Hb, PCV, MCH, MCV, and MCHC were observed following exposure of rats to lead and lead acetate. Analysis of total leucocytes count and differential leucocyte count revealed leucopenia and lymphopenia (Table 2) in PCBs-treated group. This might be due to direct toxic action of PCBs on leucopoiesis in lymphoid organs. Decrease in TLC was directly related with either their decreased production from the germinal center of lymphoid organs or increased lysis due to presence of chlorinated compounds in the body [30].

In the present study, increased AST and ALT were observed in group III and IV, which might be due to increased cell-membrane permeability or cell-membrane damage of hepatocytes caused by PCB. These findings are in accordance with toxic compounds like lead and lead acetate [31]. Among all treatment groups increase in ALP was highest in group III as compared to control group. The increase in ALP might be due to the damage of liver, kidney, and bone resulting in the liberation of ALP. Increase in ALP level observed in the present study is in agreement with the findings of Shalan et al. [31]. The mean GGT levels were decreased in group III and IV compared to control group, and this decrease was found to be an indication of hepatotoxicity and oxidative damage in the hepatocytes.

Our earlier studies proved that PCBs induce toxic effects including reproductive toxicity particularly disrupts Leydig cellular [32] and Sertoli cellular metabolic functions in both *in vivo* and *in vitro* [33]. PCB can interact with H_2_O_2_ to form hydroxyl radical, the most active form of ROS in biological system. The presence of hydroxyl radical in rat testicular cells (Sertoli, Leydig), Prostate, epididymis, liver, thyroid, kidney, and selected brain regions [34] are due to an increase in LPO with a reduction in antioxidant enzymes. PCB also inhibits testosterone biosynthesis and antioxidant enzymes in rat Leydig cells [32, 35]. Differential expression of androgen and estrogen receptors in ventral prostate of PCB exposed rats has also been studied [34]. Oxidative stress alters creatine kinase system in serum of PCB exposed rats [21]. The inhibiting effects of PCB on Leydig cell LH receptor, serum hormonal profiles of FSH, LH, testosterone, and thyroid hormone have also been studied in our previous studies.

Quercetin showed beneficial effects on liver damage by enhancing antioxidant enzyme activity and decreasing pro-oxidant effect [36]. This is due to the ability of quercetin to interact with hydroxyl, superoxide, alkoxyl and peroxyl radicals subsequently scavenging them. Quercetin supplementation led to a slight decrease in antioxidant defense in controls. This may be due to pro-oxidant effect of quercetin in normal cells. Choi et al. [37] had observed earlier that quercetin acts as a pro-oxidant in normal rats.

Insignificant change in serum total lipids and total cholesterol of rats treated with an antioxidant (quercetin) and rats treated with quercetin combined with PCBs may be due to the protective role of quercetin to prevent oxidation of the hormone-sensitive lipase which regulate lipid and cholesterol metabolism [30] while, treatment with PCBs revealed a significant decrease in serum total lipids and significant increase in serum total cholesterol. The decrease in total lipids may be due to lipolysis, via stimulation of hormone-sensitive lipase and the elevation in serum total cholesterol level could be attributed to the peroxidation of cell-membrane lipids or to the blockage of liver bile ducts, causing reduction or cessation of its secretion into the duodenum, consequently, leading to its presence in the serum resulting in cholestasis. Increase in serum total cholesterol also might be due to the mobilization of free fatty acids from the adipose tissue to blood stream and increase level of acetyl CoA, resulting in increased synthesis of cholesterol [38]. Triglycerides are fats that provide energy for the cell. Like cholesterol, they are delivered to the body's cells by lipoproteins present in the blood. A diet with a lot of saturated fats or carbohydrates will raise the triglyceride level. Increased serum triglycerides are relatively nonspecific, for example liver dysfunction resulting from hepatitis, extra hepatic biliary obstruction, or cirrhosis and diabetes mellitus are all associated with this increase [39].

In addition, serum total proteins including albumin was found to be decreased in PCB-treated rats which may be due an alternation in the intracellular protein synthesis mechanism and the level of oxidative enzymes in liver [16]. This result is in agreement with Williams and Iatropoulos [40] who reported that the decrease of protein may be attributed to reduction of serum globulin level and supports impaired immunoglobulin production. These were accompanied by a decrease of body weight which may be a result of toxicity, especially in the muscle. The results of the present study showed that treatment with PCBs after experimental period (30 days) caused a drastic reduction in the serum albumin level, which may be due to loss of protein formation from the alimentary tract, or due to decreased formation of protein in the liver (impaired ability of the liver to form albumin) [16]. Rats treated with PCBs revealed significant decrease in serum globulin and A/G ratio. This decrease may be due to the disturbance on immunoglobulin production or may be due to blocked protein synthesis. Thus leads to an increase of free amino acids and decrease in protein turnover.

Hypernatremia is usually associated with dehydration, which occurs due to decreased water level resulting in a high sodium concentration in disguise. This water loss can occur from illnesses where there is prevalence of vomiting or diarrhea, excessive sweating through exercise or fever, or from drinking fluid that has too high concentrations of salt. Too much or too little sodium can cause cells to malfunction. Lethargy, confusion, weakness, swelling, seizures, and coma are symptoms that can occur with high or low level of sodium [41]. Rapid collection of sodium can cause abnormal flow of water into or out of cells. Potassium is mostly concentrated inside the cells and sodium in the outside. The gradient, or the difference in concentration of sodium and potassium ions within the cell to that in the serum, is essential for the generation of the action potential that allows muscles and the brain to function efficiently. Chloride is the major anion (negatively charged ion) found in the ECM and in the blood. It plays a role in helping the body to maintain a normal fluid balance. Bicarbonate ion acts as a buffer to maintain the pH in blood and its levels are measured to monitor the acidity of the blood and body fluids. The acidity is affected by foods or medications that are ingested. Disturbance in the normal bicarbonate level may be due to diseases that interfere with respiratory function, kidney functions, metabolic conditions, and many more.

There are about 15 components of nonprotein nitrogenous (NPN) mostly arise from the catabolism of proteins and nucleic acids. The important components are urea, amino acids, uric acids, creatinine, creatine, and ammonia. Urea is the end product in the metabolism of protein and is formed in the liver. It appears to be elevated in blood (uremia) during diets with excess of proteins, renal diseases, heart failure, gastrointestinal hemorrhage, dehydration, or renal obstruction [41]. Uric acid and its salts are end products of the purine metabolism. With progressive renal insufficiency, there is retention of urea, creatinine, and uric acid in blood. Elevated uric acid level may be indicative of renal insufficiency and is commonly associated with gout. In the present also the elevated levels of urea and decreased levels of Uric acid and creatinine shows the renal obstruction in the PCB treated rats and it was protected in the quercetin-supplemented rats.

Glucose is a major source of energy for most cells of the body. Insulin facilitates glucose entry into the cells. Diabetes is a disease manifested by hyperglycemia; patients with diabetes type I demonstrate an inability to produce insulin. Glucose is also a key molecule in carbohydrate metabolism. It is formed as a result of the digestion of complex carbohydrates or as a result of its synthesis in the body (gluconeogenesis) [42]. The present data showed significant increase in the serum glucose of PCBs-treated rats. This may be due to the effect of PCBs on cells of pancreas which lead to low secretion of insulin hormone in turn increasing glucose in serum or may be due to liver disease or due to adrenocorticol insufficiency, anterior pituitary insufficiency and hyperthyroidism [43]. Creatinine forms as the result of degradation of creatine. Creatinine could be transformed into ATP, which is a source of high energy for the muscle cells. The creatinine production depends on the modification of the muscular mass, and it varies little, as the levels usually are very stable. Lipid content and cholesterol are abnormal in the PCBs-treated rats leading to abnormalities in the body mass. The level of the creatinine is brought back to normal by the quercetin. Bilirubin is a breakdown product of hemoglobin. It is transported from the spleen to the liver and excreted into bile. Hyperbilirubinemia results from the increase of bilirubin concentrations in serum. Causes of hyper bilirubinemia include increased hemolysis, genetic errors, neonatal jaundice, ineffective erythropoiesis, and drugs [44]. Due to the hemolysing property of PCB, the level of total bilirubin was increased in the PCB-treated group. But in the PCB+Quercetin-treated group, the total bilirubin level was equal to that of control. Quercetin induces intrinsic and extrinsic-mediated apoptosis in prostate cancer cells [45, 46]. It has electron-donating property which is due to the presence of a phenolic hydroxyl group. This property is essential for exerting antioxidant activity by scavenging free radicals [47]. Our studies proved that PCB induces oxidative stress by decreasing the activities of antioxidant enzymes in all brain regions, Kidney, Liver, thyroid, Seminal vesicle, prostate, epididymis [48, 49], and Sertoli & Leydig cells [32]. It also induced neuronal damage as well as degeneration of liver, prostate, and testicular histoarchitecture [18, 34].

Thus the present study proves that quercetin has a protective role against PCBs-induced alteration on all hematological and biochemical parameters of blood in adult rats.

## Figures and Tables

**Table 1:** 

S. no	Parameters/units	Units	Methods
1	GLUCOSE	mg/dL	Glucose oxidase
2	BUN	mg/dL	
3	UREA	mg/dL	Urease/glutamate-dehydrogenase
4	CREATININE	mg/dL	Alkaline-picrate (Jaffe)
5	URIC ACID	mg/dL	Uricase-peroxidase
6	TOTAL BILIRUBIN	mg/dL	DMSO
7	TOTAL PROTEIN	g/dL	Biuret
8	ALBUMIN	g/dL	Bromcresol green, Colorimetric
9	GLOBULIN	g/dL	Calculation
10	A G RATIO		Calculation
11	AST	IU/L	AST, pyridoxal 5-phosphate
12	ALT	IU/L	ALT, pyridoxal 5-phosphate
13	ALP	mg/dL	p-NPP method
14	GAMMA GT	mg/dL	Gamma glutamyl-3carboxy-4-nitroanilide
17	SODIUM	mmol/ L	Ion selective electrode
18	POTASSIUM	mmol/ L	Ion selective electrode
19	CHLORIDE	mmol/ L	Ion selective electrode
20	BICARBONATE	mmol/ L	Ion selective electrode
21	CHOLESTEROL	mg/dL	Cholesterol oxidase
22	TRIGLYCERIDES	mg/dL	Lipase/glyscerol
23	HDL CHOLESTEROL	mg/dL	Cholesterol oxidase
24	LDL CHOLESTEROL	mg/dL	
25	VLDL CHOLESTEROL	mg/dL	
26	T. CHOL/HDL RATIO		Calculation
27	AMYLASE	mg/dL	Maltoheptoside
28	LIPASE	mg/dL	Kinetic colorimetric, Liquid
29	CPK	mg/dL	

**Table 2:** Effect of quercetin on haematological parameters of PCBs-exposed adult rats.

S. No.	Parameters/abb	Units	Control	Quercetin	PCB	PCB+Quercetin
1	Haemoglobin (Hb)	gm/dl	13.5 ± 1.003	14.2 ± 0.521	10.5 ± 1.306^ab^	12.1 ± 0.890^c^
2	Haematocrit (PCV)	%	44 ± 4.394	45 ± 2.764	33 ± 4.389^ab^	39 ± 3.639^c^
3	Total RBC (RBC)	milli/ccmm	6.87 ± 1.140	6.60 ± 1.032	4.93 ± 0.974	5.74 ± 0.829^a^
4	Total WBC (TC)	cells/ccmm	12970 ± 2307.139	11472 ± 2640.119	9245 ± 1052.69^a^	8567 ± 570.185
5	Differential count (DC)					
	Polymorphs	%	38 ± 8.285	46 ± 7.952	53 ± 8.697	49 ± 1.600
	Neutrophils	%	57 ± 7.847	51 ± 7.948	40 ± 9.552^a^	50 ± 2.358^c^
	Eosinophils	%	4 ± 1.064	2 ± 0.557^a^	6 ± 2.290^b^	2 ± 0.307^ac^
6	Erythrocyte sedimentation rate (ESR)					
	1/2 Hour	mm	1.33 ± 0.714	1.33 ± 0.421	6.58 ± 1.200^ab^	1.83 ± 0.600^c^
	1 Hour	mm	5.0 ± 1.612	4.33 ± 1.021	15.16 ± 2.372^ab^	5.16 ± 0.872^c^
7	Platelets (Plt)	lakhs/ccmm	4.93 ± 0.961	4.44 ± 0.983	2.16 ± 0.586^ab^	4.30 ± 0.700^c^
8	Mean corpuscular volume (MCV)	fl	72.1 ± 7.180	70.6 ± 7.374	61.3 ± 3.955	70.0 ± 5.674
9	MCH	pg	22.8 ± 2.937	23.1 ± 3.673	21.3 ± 2.666	23.6 ± 2.666
10	MCHC	%	32.0 ± 1.125	32.6 ± 1.977	33.1 ± 2.257	31.8 ± 1.351

Each value represents mean ± SEM of 6 animals. Statistical significance at *P* < 0.05.

^a^Control versus others; ^b^quercetin versus PCB, PCB+quer; ^c^PCB versus PCB+quer.

**Table 3:** Effect of quercetin on general biochemical parameters of PCBs-exposed adult rats.

S. No	Parameters	Units	Control	Quercetin	PCB	PCB+Quercetin
1	Glucose	mg/dL	105 ± 5.645	97 ± 2.455^a^	138 ± 12.385^ab^	103.16 ± 4.578^c^
2	Creatinine	mg/dL	0.76 ± 0.111	0.83 ± 0.102	0.8 ± 0.270	0.75 ± 0.154
3	Total Bilirubin	mg/dL	0.3 ± 0.068	0.3 ± 0.090	0.8 ± 0.187^ab^	0.4 ± 0.084^c^

Each value represents mean ± SEM of 6 animals. Statistical significance at *P* < 0.05.

^a^Control versus others; ^b^quercetin versus PCB, PCB+quer; ^c^PCB versus PCB+quer.
